# A Retrospective Hospital-Based Study to Evaluate the Results of Extensor Tendon Injuries of the Hand, Wrist, and Forearm Treated Surgically in a Tertiary Care Hospital

**DOI:** 10.7759/cureus.65486

**Published:** 2024-07-27

**Authors:** Spurthi Sanganboina, G.S.R Hareesh, Lakshmi Meena Jasti, Purushotham G

**Affiliations:** 1 Department of Plastic Surgery, Government Medical College, Ongole, IND; 2 Department of General Surgery, Government Medical College, Ongole, IND

**Keywords:** surgical treatment, forearm, wrist, hand, extensor tendon injuries

## Abstract

Introduction

The important factors determining the treatment of extensor tendon injuries include the anatomical zone, type of injury, mode of injury, chronicity, and pathology of the adjacent tissues (principally the skin, bone, and joints). Very few studies have collectively studied the outcomes of all the wrist, forearm, and hand extensors. Hence, the major aim of this study was to evaluate the results of extensor tendon injuries of the hand, wrist, and forearm that were treated surgically.

Methodology

This was a hospital-based retrospective study done in a tertiary teaching hospital in South India. A total of 30 patients (23 males, seven females) were included in the study. All the cases of extended tendon injuries of the wrist, hand, and forearm were treated surgically, and those willing to participate were included in the study after obtaining institutional ethics committee approval.

Results

The study included 30 patients, predominantly males (76.66%). The majority were aged 31-40 years (33.33%). Occupational injuries were the most common cause (36.66%), followed by road traffic accidents (RTAs) and glass cuts (26.66%). Right-sided injuries were more frequent (56.66%), with zone VI being the most affected (43.33%). Extensor digitorum communis was the most injured tendon (40%). Various suture techniques were used, including horizontal mattress and modified Kessler’s. Complications occurred in four patients, including hematoma and surgical site infections. Functional outcomes, assessed by Miller’s Criteria, indicated extension lag and flexion loss as key recovery measures.

Conclusion

Hand function is essential for daily life activities, and optimal repair and reconstruction of extensor tendon injuries are crucial to avoid functional disability. While the present study demonstrated positive outcomes, further research with larger sample sizes and more rigorous designs is needed to validate these findings and improve treatment strategies for hand injuries.

## Introduction

The important factors determining the treatment of extensor tendon injuries include the anatomical zone, type of injury, mode of injury, chronicity, and pathology of the adjacent tissues (principally the skin, bone, and joints) [1]. The goal of any hand injury treatment is to restore form and function. Traumatic disruptions of the extensor mechanism represent a broad spectrum of injuries and are frequent because of the superficial location of tendons and concomitant injury to the bone, joints, and skin [2]. The extensor mechanism of the hand and digits is a balance between intrinsic and extrinsic forces and is easily disrupted when injured [2]. Wound debridement, rigid internal fixation of bone, repair of neurovascular structures, and skin coverage take precedence over extensor tendon repair.

Several factors play a key role in determining the course of treatment when assessing the injured hand. The major factors in this are the number and nature of damaged structures and the irreversible loss of viability of soft tissues overlying the injured tendons. The precision with which the extent of loss of viability of tissue can be determined plays an important role. The time between injury and repair, surgeon's expertise, and patient comorbidities should be considered [3].

Very few studies have examined the outcomes of all the wrist, forearm, and hand extensors in the present literature. Understanding this dearth in literature, this study was carried out to retrospectively evaluate the results of extensor tendon injuries of the hand, wrist, and forearm treated surgically in a tertiary care hospital. The objectives of the study were to comprehensively evaluate the outcomes of surgical treatment for extensor tendon injuries of the hand, wrist, and forearm. Given that the anatomical zone, type of injury, mode of injury, chronicity, and pathology of adjacent tissues (principally the skin, bone, and joints) are critical factors in determining treatment, this study aimed to address the gaps in existing research by analyzing these variables collectively. The major aim was to assess the effectiveness of surgical interventions and provide insights into the best practices for managing these complex injuries.

## Materials and methods

Study design and participants

This was a hospital-based retrospective study carried out in a tertiary teaching hospital in South India. All the cases of extended tendon injuries of the wrist, hand, and forearm were treated surgically, and those willing to participate were included in the study after obtaining institutional ethics committee approval. Consent was obtained through telephone or during review visits from all participants in this study. The Institutional Ethics Committee Government Medical College, Ongole, issued approval with reference number IEC/GMC-OGL/198/2024.

All the patients with associated skeletal injuries, injuries with massive tissue loss, and patients with both flexor and extensor tendon injuries were excluded from the study. A total of 30 patients (23 males, seven females) were included in the study. All the surgeries were performed with either interscalene or axillary blocks or general anesthesia. The anesthetist, surgeon, and instrumentation were the same in all patients.

Data sources and variables

Tourniquet control was used in every patient. All cases underwent thorough debridement. Adequate exposure was attained by raising local flaps and exploring the wounds. Tendon stumps were retrieved, and margins were resected till viable tissue was seen. Proper extension positioning of the involved digit or wrist was done according to the zones involved. The proximal and distal tendons were mobilized, and repair was done using modified Kessler’s technique or mattress suturing with Polypropylene 3-0 core sutures and 4-0 for epitenon suturing. The tourniquet was deflated, and after hemostasis was achieved, a drain was placed. Wounds were closed in layers, and a proper dressing and plaster-of-Paris (POP) splintage were given according to the zones involved. Preoperative antibiotics were given at the time of admission, and postoperative intravenous antibiotics were given for three days.

Different parameters were studied and analyzed, including total active extension regained for fingers and wrist joints, degree of extension lag in the fingers, degree of flexibility loss in the fingers, and functional recovery, which was also evaluated by the duration of return to work. Finally, the patients were asked to revisit the surgeon for follow-up at frequent intervals of three, six, 12, and 24 weeks and was assessed by Miller’s Criteria at each visit. Postoperative physiotherapy was started at the end of three weeks.

This study followed the following suture techniques for injuries involving different zones. In the zone I, conservative management was achieved through external splinting. The extensor apparatus is repaired using the horizontal mattress suture in zone II. In zone III, the extensor apparatus is repaired using the horizontal mattress suture technique. The extensor apparatus is repaired in zone IV with the figure 8 suture technique. Kessler's suture technique was modified for zones V to VIII injuries. The horizontal mattress suture technique was followed for injuries involving zone IX. The same has been tabulated in Table 1. 

**Table 1:** Types of suture techniques used for tendon repair Extensor tendon zones: Zone I: Distal to the distal interphalangeal (DIP) joint (tip of the finger); zone II: between the DIP joint and the middle phalanx; zone III: proximal interphalangeal (PIP) joint area; zone IV: proximal phalanx; zone V: metacarpophalangeal (MCP) joint area; zone VI: metacarpal bones (back of the hand); zone VII: wrist area; zone VIII: distal forearm; zone IX: proximal forearm Thumb extensor tendon zones: T zone I: distal to the interphalangeal (IP) joint of the thumb; T zone II: between the IP joint and MCP joint of the thumb; T zone III: MCP joint area of the thumb; T zone IV: first metacarpal bone area

S.no	Suture technique	Zone of repair
1	Horizontal mattress	II, III, IX
2	Modified Kessler’s	V, VI, VII, VIII, T IV
3	Figure of 8	IV

Statistical analysis

Descriptive statistics were used in the analysis of statistics to summarize details about the patients and their clinical characters. Frequencies and percentages were calculated for categorical variables, including gender, age group, cause of injury, side of injury, and zones of injury. Similarly, the evaluation of injured tendons’ distribution and complications was made through descriptive analysis. Miller's Criteria assessed functional outcomes by assessing the degree of extensor lagging and flexion loss. These analyses enabled sample distribution comprehension vis-à-vis the prevalence of different factors relating to an injury without inferential statistical tests. Correct presentation of data was ensured by conducting all data processing using appropriate statistical software.

## Results

A total of 30 patients are included in the study, of which 23 (76.66%) are males and seven (23.34%) are females. The majority of the patients, 33.33% (10), were between 31 and 40 years, followed by 26.66% (8) who were between 21 and 30 years, as shown in Table 2.

**Table 2:** Age distribution The table shows the age group distribution, the corresponding number of patients, and the percentage they contribute to the total number in the brackets

Range of age	Number of patients
<10 yrs	1 (3.33%)
11-20 yrs	7 (23.33%)
21-30 yrs	8 (26.66%)
31-40 yrs	10 (33.33%)
41-50 yrs	4 (13.33%)

**Figure 1:**
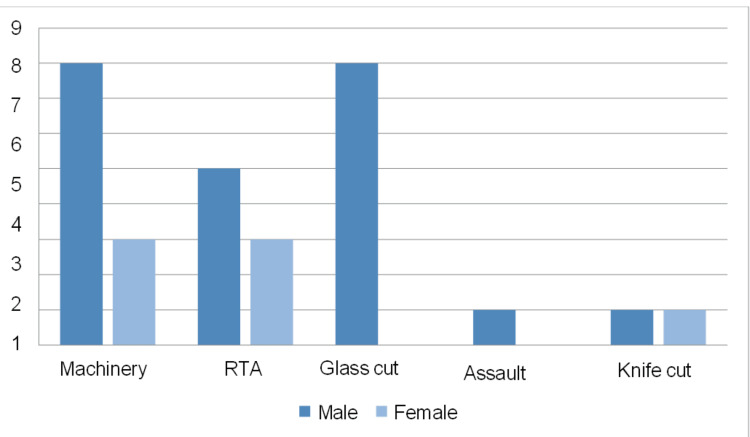
Mode of injury X-axis: Mode of injury; Y-axis: number of patients; RTAs: road traffic accidents

The most common cause of extensor tendon injuries was occupational, which was seen in 36.66 % of patients. The second most common cause was road traffic accidents (RTAs) and glass cut injuries in 26.66 % of patients. A total of 6.66% of patients had a knife-cut injury. One patient (3.33%) had an injury due to assault, as shown in Figure 1. 

The most common site of injury was noted in zone VI, accounting for 43.33% of patients, excluding the zones of injury in the thumb. The most common site of injury in the thumb was noted in zone T IV, which accounted for 10% of the total number of patients, as depicted in Table 3.

**Table 3:** Zones involved in the injuries The table shows the distribution as per the zone of injury, the corresponding number of patients, and the percentage they contribute to the total number in the brackets. Extensor tendon zones: Zone I: Distal to the distal interphalangeal (DIP) joint (tip of the finger); zone II: between the DIP joint and the middle phalanx; zone III: proximal interphalangeal (PIP) joint area; zone IV: proximal phalanx; zone V: metacarpophalangeal (MCP) joint area; zone VI: metacarpal bones (back of the hand); zone VII: wrist area; zone VIII: distal forearm; zone IX: proximal forearm Thumb extensor tendon zones: T zone I: distal to the interphalangeal (IP) joint of the thumb; T zone II: between the IP joint and MCP joint of the thumb; T zone III: MCP joint area of the thumb; T zone IV: first metacarpal bone area

Zones	Total no of patients	Patients with single-zone injury
I	1 (3.33%)	1 (3.33%)
II	2 (6.66%)	1 (3.33%)
III	1 (3.33%)	1 (3.33%)
IV	2 (6.66%)	1 (3.33%)
V	3 (10%)	2 (6.66%)
VI	13 (43.33%)	10 (33.33%)
VII	4 (13.33%)	3 (10%)
VIII	5 (16.66%)	5 (16.66%)
IX	1 (3.33%)	1 (3.33%)
T I	0	0
T II	0	0
T III	0	0
T IV	3 (10%)	2 (6.66%)

About 56.66% of patients underwent intervention within three days of injury, while 40% of patients had delayed surgical procedures after three days of injury due to delayed hospital presentation. In this study, the extensor digitorum communis (40%) is the most common injured tendon, and the next common was the extensor carpi radialis longus tendon(20%). The least common injured tendon is the extensor carpi ulnaris (3%).

A total of four patients had complications. One patient had a hematoma, which was drained. Two patients had surgical site infections, of which one of them presented with skin flap margin necrosis and the other with extension lag as a late complication. One of the other patients presented with extension lag. The two patients who presented with extension lag had zone VII injuries and were managed with physiotherapy. Functional outcomes are assessed using Miller’s Criteria, which include the degree of extensor lag and the degree of flexion loss in the affected fingers.

In the present study, the outcomes six weeks after surgery were evaluated and categorized into three distinct levels of effectiveness. It was found that 36.66% of the cases exhibited excellent results, indicating a high level of patient satisfaction and successful surgical intervention with minimal complications or issues. A larger portion, 46.66%, demonstrated good results, suggesting that while these patients experienced positive outcomes, there may have been minor complications or less optimal recovery compared to the excellent category. Lastly, 16.66% of the cases were rated as having fair results, implying that these patients had a moderate level of improvement with some notable complications or slower recovery times.

## Discussion

Hand function is crucial for maintaining independence during daily life activities. Hand injuries account for 20% of all treated injuries in an emergency department [3]. Extensor and flexor tendon systems contribute together with a complex arrangement to give a precise balance of force and positioning of the fingers; therefore, an optimal repair and reconstruction of the extensor tendon should always be attempted to avoid functional disability [4].

The mean age of the patients in the present study was 28.9 compared to 37.17, as reported by Karabeg et al. [5]. This study included 23 (76.66%) male patients and seven (23.34%) female patients, which was similar to a survey done by Karabeg et al. [5]. According to their study, 87.8% of male and 12.2% of female patients were included, which showed male preponderance. This data is consistent with epidemiological data by other authors such as Takami et al. [6] and Patillo et al. [7].

The mean age of male patients was 27.86, and female patients were 32.4 in this study; this is comparable to a study done by Karabeg et al. [5], who reported 36.19 as the mean age of the male patients and 44.20 as the mean age of the female patients. In the present study, it was observed that extensor tendon injuries of the right hand were 57% and of the left hand were 43%, which was similar to Takami et al. [6], wherein 71% of patients had an extensor tendon injury of the right hand, and 29% had an injury to the left hand.

The most common site of injury in this study was in zone VI, noted in 43% of patients, which is similar to a study done by Karabeg et al. [5], who reported zone VI as the common zone of injury. The tendons of the thumb were injured in 10% of patients, and zone T IV was the common site of injury when compared to the Karabeg et al. study, which reported 43.9% of patients with thumb extensor tendon injury in zones T III and T IV being common sites of injury. Patillo et al. [7] noticed thumb injury in 69% of their patients, which is higher compared to the present study.

According to the present study, sharp lacerations were the most common mechanism of injury in 73.3% of patients, which is comparable to the study done by Patillo et al. [7], who reported sharp lacerations in 60% of their patients [8]. Treatment consisted of primary repair of the extensor tendon injury in 56.66% of patients within the first three days following an injury and delayed primary repair in the other 40% of patients three days after the initial trauma. The patients were followed up for six months.

In our study, the total number of patients with complications was four (13.33%). One patient had a hematoma, which was drained. Two patients had surgical site infections (6.66%), with one of them presenting with skin flap margin necrosis and the other with extension lag as a late complication. One of the other patients presented with extension lag. The two patients (6.66%) who presented with extension lag had zone VII injuries, and they were managed with physiotherapy and recovered a good range of motion. These results were similar to a study by Kadah, who reported complications in 17.8% of his patients [9]. His study noted that 7.1% of his patients with postoperative infections were managed conservatively. He noticed extensor lag in 7.1% of his patients who had an injury in zones VI and VII and were managed by physiotherapy [9-11].

The final results were evaluated according to Miller’s total active motion evaluation criteria. In the present study, six weeks after surgery, excellent results were found in 36.66% of cases, good results in 46.66%, and fair results in 16.66%, which were similar to a study done by Kadah [9], who presented excellent results in 32.1% of cases, good in 42.8, and fair results in 17.8%. Outcomes following extensor tendon repair even rely upon the zone of injury involved. It is reported in the literature that poor results were observed at the extensor retinaculum level and over the fingers' dorsum. Surgical techniques evolved, and results at the extensor retinaculum level (VII) have improved, but the results of injuries at the proximal phalangeal and PIP joint levels (zones III and IV) have remained problematic [10-12].

According to studies by Lovett et al. [13] and Verdan [14], the prognosis for injuries over the proximal phalanx is worse. Elliot notes that adhesions and tenolysis often aggravate lesions over the proximal phalanx (zone 4). Zones 5 through 8 that were higher did better. However, our investigation could not identify any appreciable variations in the results across several zones [15].

In this study, a static splint was applied immediately at the surgery's end according to the zone of injury affected. Because of the patients' economic constraints, the main splinting technique used was static (100%), which corroborates with the study done by Kadah, who used static splint in 96.66% of his patients [9]. The splints were applied for a mean period of 3.23 weeks, ranging from three to four weeks, after which physiotherapy was started.

Limitations

One of the primary limitations of this study is the relatively small sample size of 30 patients, which may limit the generalizability of the findings to a broader population. The study's observational nature also means it can establish associations but not causation. The lack of a control group further limits the ability to compare outcomes and determine the efficacy of different treatment methods. Furthermore, the data relies on accurate patient recall and record-keeping, which may introduce recall or reporting biases. 

## Conclusions

In conclusion, hand function is essential for daily life activities, and the intricate balance between the extensor and flexor tendon systems underscores the importance of precise repair and reconstruction of extensor tendon injuries to prevent functional disability. The study highlights the critical need for effective surgical techniques to ensure optimal recovery and maintain hand function. Despite the promising outcomes observed, the relatively small sample size and the study's observational nature limit the generalizability of the findings. Future research with larger sample sizes, control groups, and more rigorous designs is necessary to validate these results and explore potential causal relationships. Such studies would provide a deeper understanding of the most effective treatment methods and further enhance patient care and quality of life following hand injuries.
